# Host Gene Expression Profiling and *In Vivo* Cytokine Studies to Characterize the Role of Linezolid and Vancomycin in Methicillin-Resistant *Staphylococcus aureus* (MRSA) Murine Sepsis Model

**DOI:** 10.1371/journal.pone.0060463

**Published:** 2013-04-02

**Authors:** Batu K. Sharma-Kuinkel, Yurong Zhang, Qin Yan, Sun Hee Ahn, Vance G. Fowler

**Affiliations:** 1 Division of Infectious Diseases, Department of Medicine, Duke University Medical Center, Durham, North Carolina, United States of America; 2 Duke Clinical Research Institute, Durham, North Carolina, United States of America; University Hospital Münster, Germany

## Abstract

Linezolid (L), a potent antibiotic for Methicillin Resistant *Staphylococcus aureus* (MRSA), inhibits bacterial protein synthesis. By contrast, vancomycin (V) is a cell wall active agent. Here, we used a murine sepsis model to test the hypothesis that L treatment is associated with differences in bacterial and host characteristics as compared to V. Mice were injected with *S. aureus* USA300, and then intravenously treated with 25 mg/kg of either L or V at 2 hours post infection (hpi). *In vivo* alpha-hemolysin production was reduced in both L and V-treated mice compared to untreated mice but the reduction did not reach the statistical significance [*P* = 0.12 for L; *P* = 0.70 for V). PVL was significantly reduced in L-treated mice compared to untreated mice (*P* = 0.02). However the reduction of *in vivo* PVL did not reach the statistical significance in V- treated mice compared to untreated mice (*P* = 0.27). Both antibiotics significantly reduced IL-1β production [*P* = 0.001 for L; *P* = 0.006 for V]. IL-6 was significantly reduced with L but not V antibiotic treatment [*P*<0.001 for L; *P* = 0.11 for V]. Neither treatment significantly reduced production of TNF-α. Whole-blood gene expression profiling showed no significant effect of L and V on uninfected mice. In *S. aureus*-infected mice, L altered the expression of a greater number of genes than V (95 vs. 42; *P* = 0.001). Pathway analysis for the differentially expressed genes identified toll-like receptor signaling pathway to be common to each *S. aureus*-infected comparison. Expression of immunomodulatory genes like *Cxcl9, Cxcl10, Il1r2, Cd14* and *Nfkbia* was different among the treatment groups. Glycerolipid metabolism pathway was uniquely associated with L treatment in *S. aureus* infection. This study demonstrates that, as compared to V, treatment with L is associated with reduced levels of toxin production, differences in host inflammatory response, and distinct host gene expression characteristics in MRSA sepsis.

## Introduction

*Staphylococcus aureus* is a leading cause of nosocomial and community-acquired infections [1], [2]. An emerging body of evidence suggests that specific bacterial toxins are involved in the pathogenesis of a variety of MRSA infections [3]–[6]. For example, alpha-hemolysin is a potent cell lysing agent, thought to induce the production of IL-1β, IL-6, and IL-8, and TNF-α [3]. In addition, the Panton-Valentine Leukocidin (PVL), a phage-associated bicomponent leukocidin, is a well-recognized virulence factor in *S. aureus*. PVL lyses neutrophils and induces the release of proinflammatory cytokines from these cells [7], [8]. Its presence in *S. aureus* has been associated with a number of clinical syndromes, including necrotizing *S. aureus* pneumonia [9]–[12], necrotizing fasciitis [13], osteomyelitis [14], and induces the release of proinflammatory cytokines from neutrophils [7], [8].

Significant evidence suggests that the interruption of bacterial toxin synthesis by protein-synthesis inhibitors may reduce toxin production and potentially improve clinical outcome [15]–[18]. It has been shown that linezolid (L), but not vancomycin (V), is a potent inhibitor of protein synthesis [18]–[20]. Thus, evidence supports the hypothesis that linezolid’s inhibition of bacterial protein synthesis can influence the clinical course of toxin-mediated infections.

In contrast to cell-wall active agents, the majority of which induce bacterial toxin production [18], [21], [22], L specifically down-regulates the production of a variety of virulence toxin genes, including PVL and alpha hemolysin, even in its sub-inhibitory concentrations [20], [22], [23]. These findings suggest that these two classes of antibiotics may have significantly different effects on the expression of bacterial toxin genes. However, despite previous efforts [24]–[26], the full impact of L on the modulation of the host-pathogen interaction in MRSA sepsis is still unknown. The current study is designed to address this gap by simultaneously evaluating the influence of two broadly different classes of antibiotics (L and V) on both the host and the pathogen. Although the host gene expression profiling has been previously used to study the host pathogen interaction in infections [27], [28] and in non-infectious conditions like cancer [29]–[31], none of the studies have used this technique to compare and contrast the effect of antibiotics on host gene expression. In the current study, we test the hypothesis that L treatment elicits a different gene expression profile compared to V treatment in MRSA sepsis. To do this, we evaluated and compared the impact of L and V on both host and pathogen gene expression using a well-characterized strain of CA-MRSA in a murine sepsis model of infection.

## Materials and Methods

### *S. aureus* Strain and Culture

*S. aureus* USA300 was obtained from NARSA (Network on Antimicrobial Resistance in *Staphylococcus aureus*). For preparation of *S. aureus* for injection, an overnight bacterial culture of *S. aureus* was diluted with fresh tryptic soy broth (TSB) (Becton Dickinson, Sparks, MD) and incubated (37°C) with aeration to log-phase growth [optical density at wavelength 600 nm (OD 600) of 1.0] [32]. *S. aureus* was harvested by centrifugation, rinsed, and re-suspended in PBS.

### Antimicrobial Agents

Linezolid was obtained from Pfizer Inc. (Groton, CT). Vancomycin was purchased from Sigma-Aldrich Chemical (St. Louis, MO).

### Animals

A/J mice (Jackson Laboratory, Bar Harbor, Maine), 8 to 10 weeks old and weighing approximately 18 g (16 to 20 g) were used in this study. Mice were housed in sterile microisolator cages (5 per cage) with sterile bedding, feed, and acidified water. All animal experiments were carried out in strict accordance with the recommendations of NIH guidelines, the Animal Welfare Act, and US federal law. All animal procedures were approved by the Institutional Animal Care and Use Committee (IACUC) of Duke University (IACUC number: #1890907) which has been accredited by the Association for Assessment and Accreditation of Laboratory Animal Care (AAALAC) International.

### Inoculum

Mice were challenged with *S. aureus* via intraperitoneal (i.p.) administration with 6×10^6^ CFU/g of *S. aureus* USA300 (0.2 ml), then intravenously injected with 25 mg/kg L or V dissolved in 0.2 ml of pyrogen free distilled water 2 hours post-infection (hpi) [33], [34]. To mimic the natural course of *S. aureus* infection in humans, which typically arises from a primary focus of infection and disseminates to other sites, we employed an i.p. route of infection in our animal model [35]. The 2 hpi time point was chosen to reflect the fact that bacteria are demonstrable in murine bloodstream within 2 hour post i.p. infection [35].

### Quantitative Culture

Blood and kidney samples were obtained from infected mice (n = 5 in each group) 24 hpi, which were serially diluted and plated on TSA agar plates at 37°C to quantify CFU. Kidneys collected from euthanized animals were homogenized in 1X PBS and diluted 10 fold serially. Blood was collected via cardiac puncture. Tissue homogenates and blood were serially 10-fold diluted in sterile 1X PBS. The serial dilutions were plated in Tryptic Soy Agar (TSA) plates and incubated (37°C, overnight) to count the number of colony forming units (CFU) of *S. aureus*. Finally the number of bacteria in blood and kidney were expressed as CFU/ml and CFU/g respectively. Statistical significance of the comparison was tested by using F-test.

### Production of Antibody to PVL

The gene encoding the PVL toxin, *lukF*, was cloned into an overexpression his-tag fusion vector (pET100/D-TOP; Invitrogen Co.), as described elsewhere [36], and purified according to the manufacturer’s instructions. Two rabbits (Rockland Immunochemicals, Inc., PA, USA) were used for antibody production by three times boosting with the LukF recombinant protein mixed in an equal volume of incomplete Freund’s adjuvant 1 week apart. Serum was obtained 45 days after the initial dose. Polyclonal antibody efficiency against PVL has been successfully tested by Western blotting (data not shown).

### Enzyme Linked Immunosorbent Assay (ELISA)

*In vivo* levels of *S. aureus* toxins (alpha-hemolysin and PVL) and cytokines in mouse serum were determined by ELISA. A 96-well plate (BD Falcon, Franklin, NJ) was coated with 10 µg/ml polyclonal antibody against rabbit in 1X PBS overnight at 4°C, after which the wells were washed and blocked with 5% bovine serum albumin in PBS. Serum was added to the wells and incubated at RT for 1 hour. Primary antibodies specific for alpha-hemolysin (Sigma-Aldrich, St. Louis, MO) or PVL (Rockland Immunochemicals, Inc.) were added to the supernatant, followed by anti-rabbit IgG horseradish-peroxidase conjugate (Sigma-Aldrich, St. Louis, MO), 2,2′-azino-bis(3-ethylbenzothiazoline-6-sulfonic acid). After 20 min, absorbance was read at 405 nm. For the measurement of cytokine, serum was separated from blood obtained by intracardiac puncture. Protein concentrations were determined by bicinchoninic acid method kit (Pierce, Rockford, IL). Equal amounts of protein for each sample were used to measure the levels of interleukin-1β, IL-6, and tumor necrosis factor alpha (TNF-α) using ELISA (Duo kit; Invitrogen) following the manufacturer’s instructions. The serum levels of cytokines were expressed as pg/ml of serum. The statistical significance between the experimental groups was studied using unpaired t-test.

### Microarray Using Whole Blood RNAs

Total RNA was prepared from mouse blood using the Mouse RiboPure Blood RNA isolation kit (Ambion, Austin, TX) following the manufacturer’s instruction. Globin mRNA was removed from whole blood RNA samples using the Globinclear kit (Ambion, Austin, TX). A total of 35 samples passed the quality criteria of the Agilent Bioanalyzer and were used for microarray analysis. Since the total RNA yield of many samples was low, one round of linear amplification was performed for all samples using the MessageAmp Premier kit (Ambion, Austin, TX). RNA integrity numbers were calculated for all samples and found to be within tolerance limits. Affymetrix GeneChip® Mouse Genome 430 2.0 Arrays were used (Affymetrix, Santa Clara, CA). The biotin-labeled cDNA was hybridized to the arrays for 16 hours at 45°C following the manufacturer’s instruction. The arrays were then washed and labeled with streptavidinphycoerythrin (strep-PE), and the signal was amplified using biotinylated antistreptavidin followed by another round of staining with strep-PE. Washing and staining were performed on the Affymetrix fluidics station according to the recommended fluidics protocol. Amplification and microarray hybridization were performed at the Duke University Microarray Core. Labeled gene chips were scanned using an Affymetrix Genechip Scanner 7G (Affymetrix, Santa Clara, CA). This array contains 45,101 probe sets to analyze the expression level of over 39,000 transcripts and variants from over 34,000 well characterized mouse genes. The microarray data have been deposited in the NCBI GEO and are accessible through GEO series accession no GSE38531.

### Microarray Data Analysis

Gene expression data were imported into Partek Genomics Suite 6.5 (Partek, St Louis, Mo) as CEL files using default parameters. Raw data were preprocessed, including background correction, normalization, and summarization using robust multiarray average analysis, and expression data were log2 transformed. Differential expression analysis for the whole blood cells was performed using 1-way analysis of variance (either infection status or drug treatment alone). Gene lists were created using a cutoff of *P*<0.05, 2-fold change, although second-level analysis using a false-discovery rate of <0.05, 2-fold change was also performed. Hierarchical clustering (heat map image) was also performed using Partek Genomics suite.

### Pathway Analysis

Pathway analysis for functional annotation of the genes was performed by GATHER KEGG pathway analysis (http://gather.genome.duke.edu/) [37]. The significance of the association between the data set and the KEGG pathway was measured in two ways: 1) Fischer’s exact test to calculate a *P*-value and 2) calculating the ratio of the pathway-associated genes in the experimental data to the total number of genes in that pathway.

## Results

### Effect of Antibiotics on Bacterial Load in Kidney and Blood

**Figure 1:**
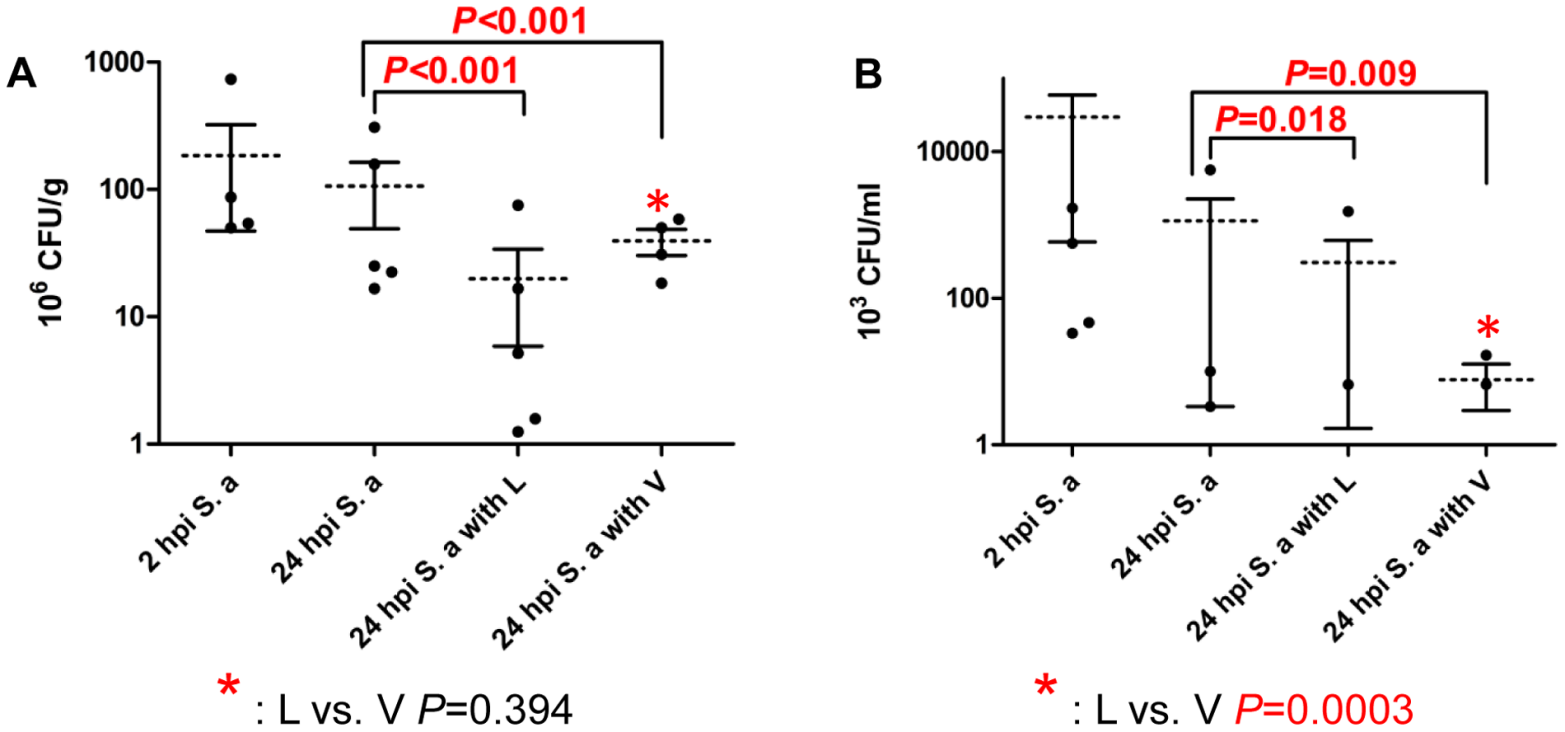
Effect of antibiotics on bacterial load in A/J mice. The bacterial loads in the (A) Kidney and (B) Blood from A/J mice after intraperitoneal infection with *S. aureus* (6×10^6^ CFU/g, strain USA300), and treatment with linezolid (L) or vancomycin (V) (25 mg/kg). Infected mice were sacrificed as follows: 2 hpi *S. aureus*; 24 hpi *S. aureus*; 24 hpi *S. aureus* with linezolid or vancomycin. Each symbol represents one mouse. *P-*values were obtained by F-test. The error bars correspond to the standard error of mean (SEM) and the dashed line correspond to the mean value. Values of *P*<0.05 were considered significant.

To characterize the bacteriological effect of L and V, we measured the tissue burden of *S. aureus* USA300 in A/J mice at 24 hours post-infection (hpi). Compared to untreated mice, bacterial loads in kidney (Figure 1A) and blood (Figure 1B) were significantly reduced in mice treated with either L or V. Bacterial counts were similar from the kidney in L and V-treated mice (19.93 × 10^6^ cfu/gm *vs.* 39.38 × 10^6^ cfu/gm; *P* = 0.394), but significantly lower from blood in V-treated mice (308 × 10^3^ cfu/ml *vs.* 7.778 10^3^ cfu/ml; *P* = 0.0003) (Figure 1).

### Effect of Antibiotic on *In Vivo S. aureus* Toxins and Cytokines Production in Serum

**Figure 2:**
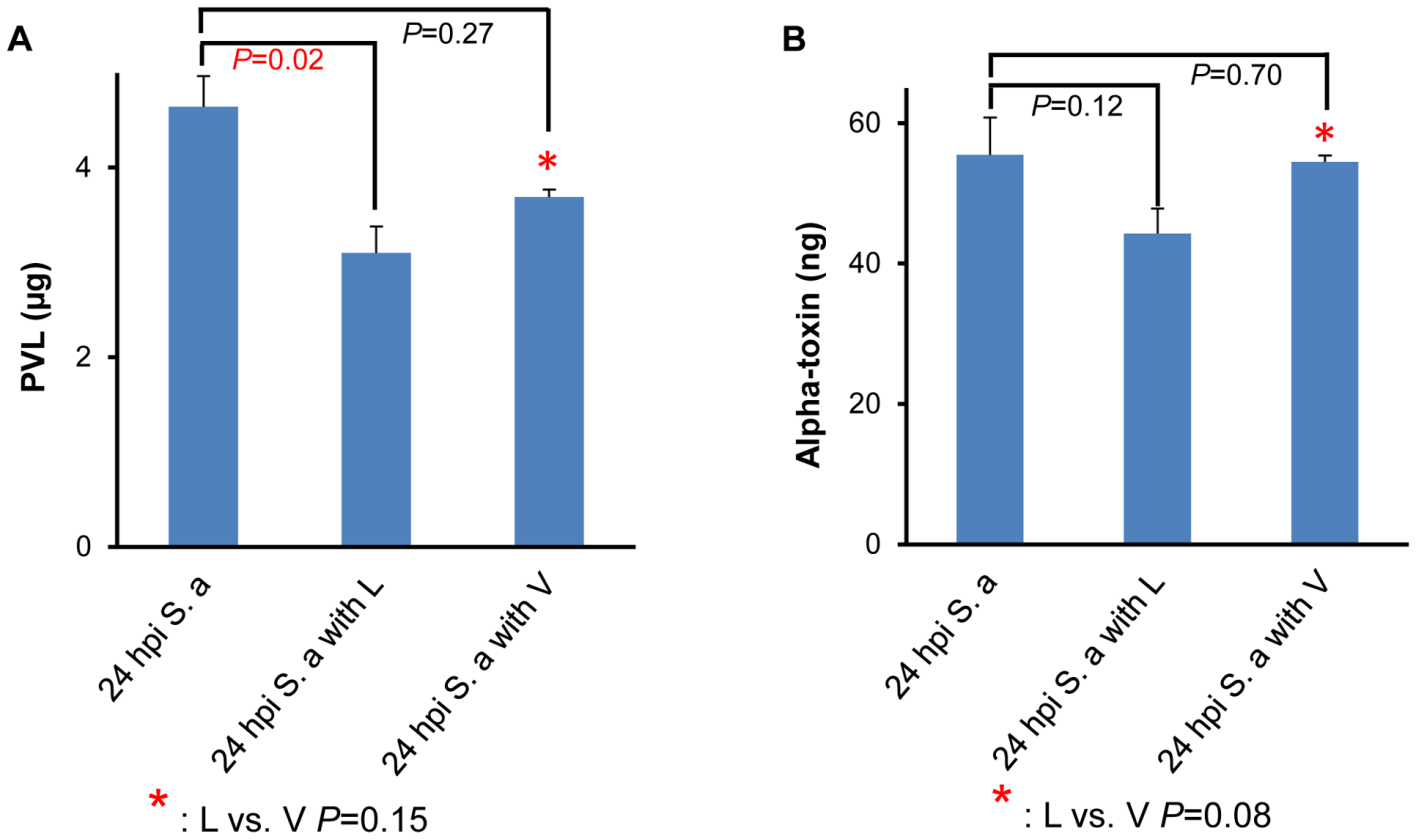
Effect of antibiotics on *in vivo* toxin production. *In vivo* level of (A) Panton Valentine Leukocidin (PVL, µg) (B) and Alpha-toxin (ng) in the serum of A/J mice after 24 h of intraperitoneal (ip) infection with *S. aureus* (6×10^6^ CFU/g, strain USA300), and treatment with linezolid or vancomycin (25 mg/kg) at 2 h was measured using ELISA. *P-*value was obtained by unpaired t-test. The error bar corresponds to the standard error of mean (SEM). Values of *P*<0.05 were considered significant.

To study the effect of toxin production suppression in staphylococcal sepsis, we evaluated the effect of L and V on *in vivo* concentration of PVL and alpha-hemolysin in mouse serum. Compared to untreated controls, L but not V was associated with significant reduction in PVL production (*P* = 0.02 in L vs. *P* = 0.27 in V) (Figure 2A). Mice treated with L and V did not differ significantly in reduction of PVL production (*P* = 0.15). Neither L nor V treatment resulted in a statistically significant difference on alpha-hemolysin production compared to the untreated controls (*P* = 0.12 in L and *P* = 0.70 in V). (Figure 2B). Despite the fact that V treatment significantly lowered the bacterial counts in blood compared to L treatment (Figure 1B), the level of both PVL and alpha-hemolysin was higher in V treated serum compared to that of L treated.

**Figure 3:**
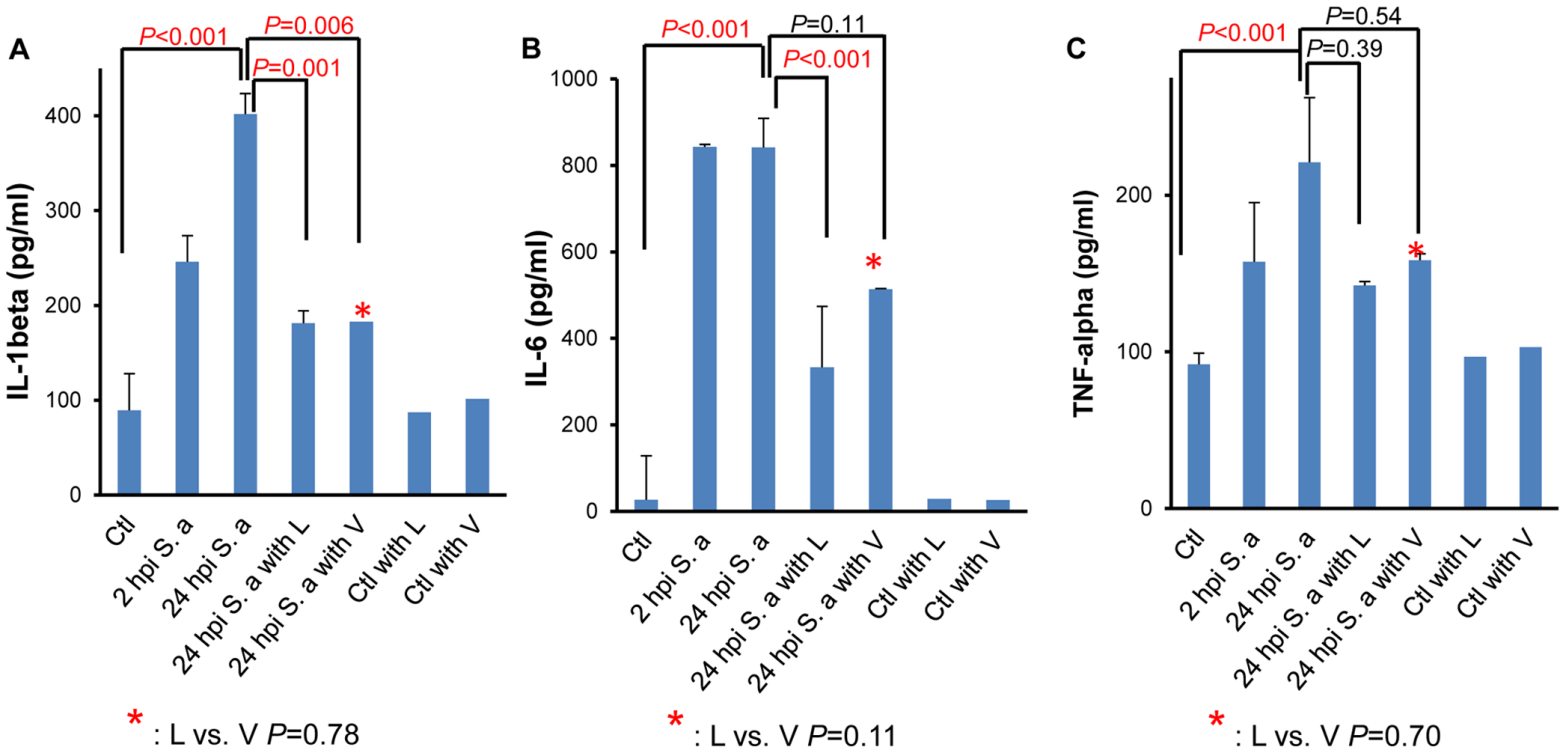
Effect of antibiotics on pro-inflammatory cytokines. Level of (A) IL-1beta (pg/ml), (B) IL-6 (pg/ml), and (C)TNF-alpha (pg/ml) from the serum of A/J mice without infection, with 2 hours post infection (hpi) with *S. aureus* (6×10^6^ CFU/g, strain USA300), and 24 hpi with and without antibiotic treatment as labeled was measured using ELISA. *P-*value was obtained by unpaired t-test. The error bar corresponds to the standard error of mean (SEM). Values of *P*<0.05 were considered significant.

To study the relative immunomodulatory effects of L and V treatment, we next measured the cytokine levels in mouse serum at 24 hpi using ELISA. Compared to uninfected controls, cytokine production was significantly increased at 24 hours following *S. aureus* infection compared to uninfected controls (*P*<0.001). Both antibiotics significantly reduced IL-1β production compared to untreated group [*P* = 0.001 in L and *P* = 0.006 in V] and there was no statistically significant difference between the L and V treatment groups (*P* = 0.78) (Figure 3A)]. Although treatment with L, but not V, resulted in a statistically significant reduction of IL-6 as compared to untreated controls [*P*<0.001 in L and *P* = 0.11 in V], the differences in IL6 production between the two treatment groups did not differ significantly (*P* = 0.11) (Figure 3B). Neither L nor V treatment resulted in statistically significant reduction in production of TNF-α (Figure 3C).

### Effect of Antibiotic on Host Gene Expression Pattern

**Figure 4:**
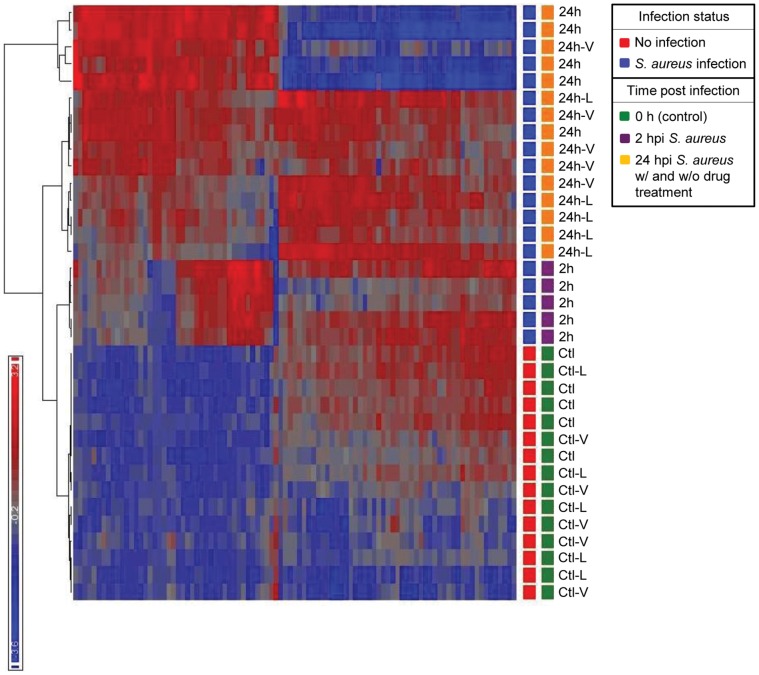
Unsupervised hierarchical clustering analysis of mouse blood microarray data. Each colored row in the heat map represents the gene expression value for a probe and each column represents a sample. The color conventions are as follows: red indicates over-expressed transcripts, blue represents under-expressed transcripts. Time of analysis post-infection are marked as follows: green rectangles indicate 0 hpi, purple rectangles indicate 2 hpi, and orange rectangles indicate 24 hpi (with and without antibiotic treatment).

To ascertain that the different treatment groups (e.g. uninfected, *S. aureus* infected, *S. aureus* infected with L treatment, and *S. aureus* infected with V treatment) can be separated into several distinct clusters based on their gene expression profiles, we next conducted an unsupervised hierarchical clustering using the gene expression data from 35 microarrays. As seen in the Figure 4 heat map, distinct clustering is clearly evident based on infection status (Red squares: uninfected, Blue squares: *S. aureus* infected) and the infection time-course [Green squares: 0 hpi, Purple squares: 2 hpi, and Orange squares: 24 hpi (with or without antibiotic treatment)].

**Figure 5:**
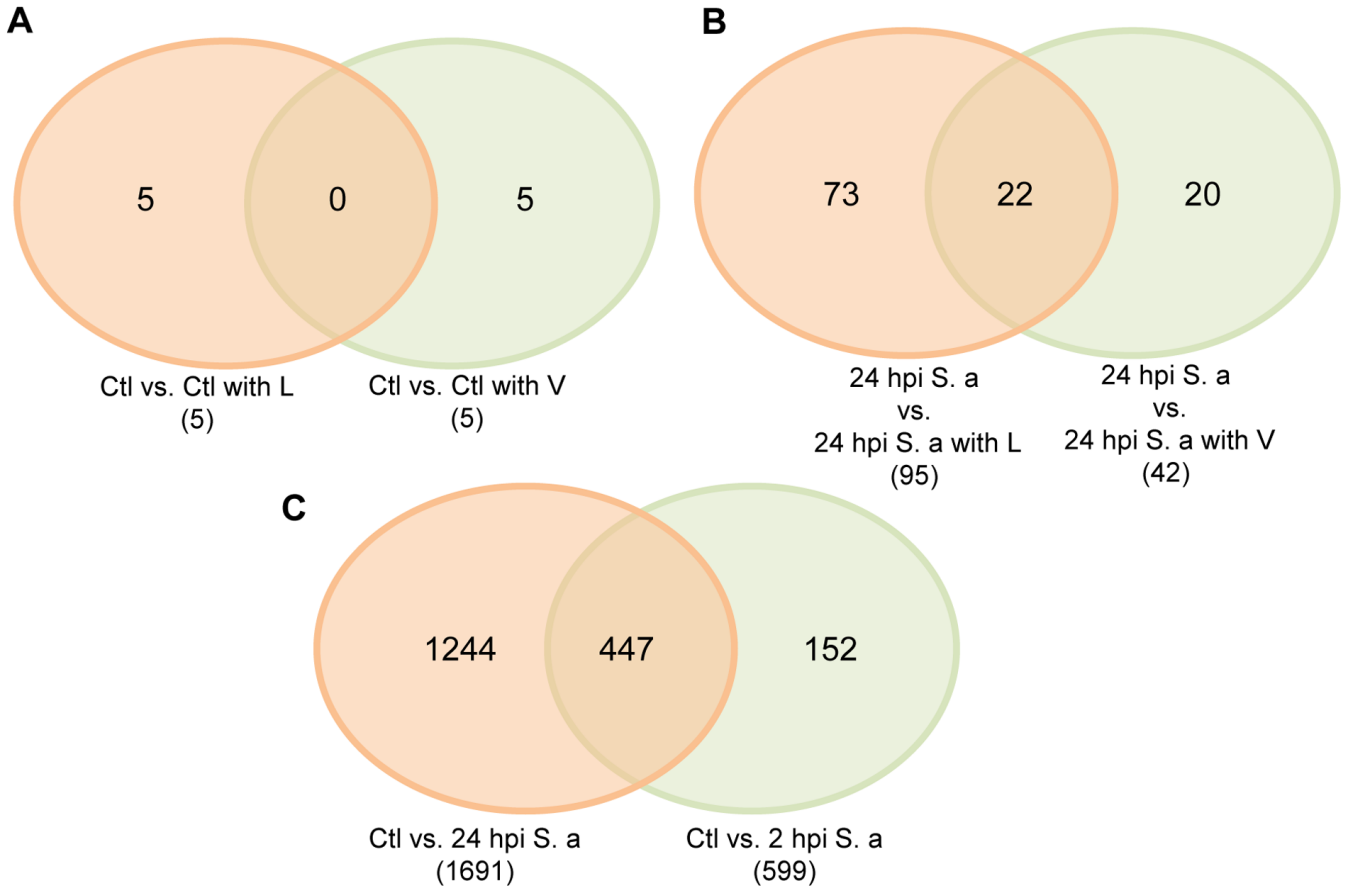
Comparisons of differentially expressed genes in mice with the appropriate treatment. (A) Effect of linezolid and vancomycin on host, (B) Effect of linezolid and vancomycin on host with *S. aureus* infection, and (C) Effect of *S. aureus* infection on host immune response. Venn diagrams indicate the number of genes unique to each comparison pair and the number of genes common to each comparison pair (overlapped).

To address the antibiotic-specific differences in gene expression, we identified the number of genes uniquely expressed in response to *S. aureus* infection, infection with L treatment, and infection with V treatment (Figure 5). When the drug-alone treatment group (L or V) was compared with uninfected/untreated group (controls), only five genes were differentially expressed solely in each group. This finding suggests that treatment with either L or V have a minimal impact on host gene expression itself and each drug shows a unique expression profile without any overlap when administered in an uninfected host (Figure 5A). Within the *S. aureus*-infected host, L treatment significantly affected more genes (95 genes) compared to V treatment (42 genes) (*P* = 0.0013). An additional 22 genes were affected by both antibiotics, indicating some overlap in the expression response induced by L or V in *S. aureus*-infected host (Figure 5B). A comparison of *S. aureus* infection dynamics shows a considerably higher number of genes differentially expressed at 24 hpi compared to 2 hpi (1691 at 24 hpi vs. 599 at 2 hpi). Around 74% of genes (447 of 599) differentially expressed at 2 hpi were also differentially expressed at 24 hpi (compared to uninfected controls) (Figure 5C). The details of all the genes differentially expressed in each comparison groups are summarized in Supplemental Table S1.

### Effect of Antibiotic on Pathways of Differentially Expressed Genes

To correlate the significance of differentially expressed genes in terms of its biological relevance, we next studied pathway associations for the differentially expressed genes in each comparison group in mice using Kyoto Encyclopedia of Genes and Genomes (KEGG). Pathways significantly associated with the differentially expressed genes are summarized in Table 1. Toll-like receptor signaling pathway (*Atm, Ccl3, Cd14, Cxcl10, Cxcl9, Ikbkb, Il6, Jun, Lbp, Ly96, Mapk11, Mapk13, Myd88, Nfkbia, Stat 1, Tirap, Tlr2, and Tlr4*) was the most frequently identified pathway common to all comparison groups studied other than Control L vs. Control V group. MAPK signaling pathway (*Casp1, Casp11, Casp3, Casp7, Cd14, Dusp1, Dusp14, Fas, Fgf13, Gadd45g, Gng12, Ikbkb, Il1r2, Jun, Map3k1, Map3k4, Map3k8, Map4k1, Map4k2, Mapk11, Mapk13, Mef2c, Pak1, Ppp3cc, Prkacb, Prkcb1, Rapgef4, Rasgrp1, Rasgrp4, Rps6ka5, Rras2, Tgfbr2, and Tnfrsf1a*) was common between Control vs. 24 h *S. aureus* infection and 24 h *S. aureus* infection vs. 24 h *S. aureus* L comparisons.

**Table 1:** Kyoto Encyclopedia of Genes and Genomes (KEGG) pathway analysis of the differentially expressed genes in each comparison groups.

Comparison groups	Pathways	p-value*	Genes
	mmu04610: Complement and coagulation cascades	<0.0001	*C1qa, C1qb, C1qg, C3, C4, Cfi, Serpina1a, Serpina1b, Serpinc1*
	mmu00361: Gamma-hexachlorocyclohexane degradation	<0.0001	*Cyp2c29, Cyp2d9, Cyp2e1, Cyp3a11, Pon1*
**Control linezolid vs. Control vancomycin**	mmu00271: Methionine metabolism	0.0001	*Bhmt, Cth, Mat1a*
	mmu00071: Fatty acid metabolism	0.0002	*Adh1, Cyp2c29, Cyp2d9, Cyp2e1, Cyp3a11*
	mmu00380: Tryptophan metabolism	0.0008	*Cyp2c29, Cyp2d9, Cyp2e1, Cyp3a11, Tdo2*
	mmu00910: Nitrogen metabolism	0.007	*Car3, Cth*
	mmu00450: Selenoamino acid metabolism	0.01	*Cth, Mat1a*
	mmu00031: Inositol metabolism	0.01	*Aldh6a1*
**24 h ** ***S. aureus*** ** linezolid vs.** **24 h ** ***S. aureus*** ** vancomycin**	mmu04620: Toll-like receptor signaling pathway	0.001	*Cxcl10, Cxcl9, Stat1*
	mmu04060: Cytokine-cytokine receptor interaction	0.01	*Cxcl10, Cxcl9, Il1r2*
	mmu00252: Alanine and aspartate metabolism	0.02	*Ass1*
**24 h ** ***S. aureus*** ** vs. 24 h** ***S. aureus*** ** linezolid**	mmu04620: Toll-like receptor signaling pathway	0.002	*Cd14, Nfkbia*
	mmu00561: Glycerolipid metabolism	0.009	*Lipg*
	mmu04010: MAPK signaling pathway	0.01	*Cd14, Il1r2*
**24 h ** ***S. aureus*** ** vs. 24 h** ***S. aureus*** ** vancomycin**	mmu04620: Toll-like receptor signaling pathway	0.003	*Cxcl10, Nfkbia*
	mmu05040: Huntington’s disease	0.009	*Tgm2*
**Control vs. 24 h ** ***S. aureus***	mmu04060: Cytokine-cytokine receptor interaction	0.0001	*Blr1, Bmp2, Ccl17, Ccl3, Ccr1, Ccr5, Ccr6, Ccr7, Csf2ra, Csf2rb2, Cxcl10, Cxcl2, Cxcl9, Cxcr4, Fas, Ifng, Il13ra1, Il15ra, Il18rap, Il1r2, Il1rap, Il28ra, Il6, Il7r, Il8rb, Kit, Ltb, Ltbr, Met, Osm, Tgfbr2, Tnfrsf13b, Tnfrsf13c, Tnfrsf1a, Tnfrsf5, Tnfrsf7*
	mmu04010: MAPK signaling pathway	0.0006	*Casp1, Casp11, Casp3, Casp7, Cd14, Dusp1, Dusp14, Fas, Fgf13, Gadd45g, Gng12, Ikbkb, Il1r2, Jun, Map3k1, Map3k4, Map3k8, Map4k1, Map4k2, Mapk11, Mapk13, Mef2c, Pak1, Ppp3cc, Prkacb, Prkcb1, Rapgef4, Rasgrp1, Rasgrp4, Rps6ka5, Rras2, Tgfbr2, Tnfrsf1a*
	mmu04080: Neuroactive ligand-receptor interaction	0.001	*Calcrl, F2rl1, Fpr1, Gpr35, Ltb4r1, P2ry1, P2ry10, Ptger3, Vipr1*
	mmu04620: Toll-like receptor signaling pathway	0.001	*Atm, Ccl3, Cd14, Cxcl10, Cxcl9, Ikbkb, Il6, Jun, Lbp, Ly96, Mapk11, Mapk13, Myd88, Nfkbia, Tirap, Tlr2, Tlr4*
	mmu00190: Oxidative phosphorylation	0.003	*Cox7c*
	mmu00030: Pentose phosphate pathway	0.01	*G6pd2, G6pdx, Gpi1, Pgd, Prps2, Tkt*

* : p-value is calculated by using Fischer’s exact test calculating the ratio of the pathway-associated genes in the experimental data to the total number of genes in that pathway.

When looked into further details for the genes involved in immune modulation, genes like *Cxcl9, Cxcl10* and *Il1r2* were differentially expressed between 24 h *S. aureus* L vs. 24 h *S. aureus* V. *Cxcl9* and *Cxcl10* were up-regulated in 24 h *S. aureus* L and *Il1r2* was down-regulated. Also *Cd14*, *Nfkbia*, and *Il1r2* were differentially expressed between 24 h *S. aureus* vs. 24 h *S. aureus* L and all three genes were down-regulated in 24 h *S. aureus* L. In addition, *Nfkbia* was down-regulated in 24 h *S. aureus* V with a significantly different expression between 24 h *S. aureus* and 24 h *S. aureus* V (Table 1 and Supplemental Table S1).

Glycerolipid metabolism pathway was significantly associated with 24 h *S. aureus* infection vs. 24 h *S. aureus* L comparison group while Huntington’s disease pathway was only associated with 24 h *S. aureus* infection vs. 24 h *S. aureus* V comparison. There was no overlap in pathways within the Control L vs. Control V groups. Similarly, comparisons involving *S. aureus* infection (24 h *S. aureus* L vs. 24 h *S. aureus* V, 24 h *S. aureus* vs. 24 h *S. aureus* L, 24 h *S. aureus* vs. 24 h *S. aureus* V and Control vs. 24 h *S. aureus*) also yielded no common gene expression pathways.

## Discussion

Despite several recent studies of antibacterial agents possessing immunomodulatory effects [20], [23], [38]–[45], the precise mechanism and effects of these antibiotics in host gene expression and immunomodulation in MRSA infection is unknown. This present study sought to understand the impact of L on the host-pathogen interaction during MRSA infection in comparison with V. Using a murine model of *S. aureus* infection, we determined that L and V induced differential production of bacterial toxins and host cytokines, differences in host gene expression, and differences in immunomodulators during MRSA bloodstream infection.

Various studies have shown that L suppresses toxin production, whereas the cell wall active antibiotics had either no effect or enhanced the production of extracellular virulence factors [18], [21]–[23], [46]–[48]. For example, Stevens et al [49] showed that L significantly reduced production of TSST even in the setting of high bacterial counts. The results of our *in vivo* study substantiate these previous findings showing more pronounced effect of L in *in vivo* production of PVL and alpha hemolysin compared to V. Collectively, these data provide the evidence that protein-synthesis inhibitors such as L that act on a ribosomal level are more effective toxin suppressors and could be more effective in treating MRSA sepsis.

Antibiotic including L and V are known to have immunomodulatory effects during *S. aureus* infection [20], [23], [38]–[45]. Consistent with previously reported findings [45], [50]–[54], treatment with both L and V reduced proinflammatory cytokine production in the serum of *S. aureus* infected mice, albeit by different mechanisms (Figure 3). Evidence from previous studies suggests that the network of inflammatory cytokines and chemokines play a major role in mediating, amplifying, and perpetuating inflammation [55], [56]. Findings from two recent studies have suggested that PVL treatment of neutrophils results in elevated gene expression and protein production of proinflammatory cytokines including IL-6, IL-8 and TNF-a [7], [8]. It has also been shown that L inhibits MRSA growth and suppresses the production of toxins *in vitro* [18], [19] and potentiates the susceptibility of *S. aureus* to human neutrophils [20]. Thus the pronounced effect of L on mice could be due to its two-pronged effect –1) direct effect of L in protein synthesis of bacterial toxins including PVL; and 2) indirect effect on toxin-mediated cytokine production during *S. aureus* infection. Although the role of PVL in *S. aureus* infections is highly controversial, there is evidence that PVL is associated with severe disease in community acquired pneumonia (CAP) caused by *S. aureus* both in clinical reports [9], [10] and in some [11], [12], but not all [57]–[60], *in vivo* model systems. Taken together, these findings suggest that L’s effect on *S. aureus* infected patients is complex and due at least in part to its effect on both bacterial protein and host cytokine production.

The murine model of host gene expression profiling has been widely used to assess the host pathogen interaction [27], [28] as well as in non-infectious conditions like radiation exposure and breast cancer [29]–[31]. In this study, we have described the drug-specific gene expression profiles in mice by comparing the response of *S. aureus* infection with and without L or V treatment. In consistent with previously reported findings [61], [62], we found that cytokine-cytokine receptor interactions is an important pathway in the murine response to *S. aureus* infection (Table 1). Similar gene expression-based analyses of the human response to bacterial infection have also revealed the importance of cytokine-cytokine receptor interactions; TLR signaling; MAPK signaling; Jak-STAT signaling; focal adhesion; and complement and coagulation cascades [63]–[65], three of which (Cytokine-cytokine receptor interactions, TLR signaling and MAPK signaling) were significantly associated with *S. aureus* infection in this study.

We have noticed that *Nfkbia*, a hallmark of the inflammatory response [66] was down-regulated in both treatment groups (24 h *S. aureus* L and 24 h *S. aureus* V) compared to untreated controls (Table 1 and Supplemental Table S1). This is in agreement with the fact that both L and V treatment reduce inflammation compared to untreated controls. Interestingly, in 24 h *S. aureus* L treatment, *Cxcl9* and *Cxcl10* were up-regulated whereas *Il1r2* was down-regulated compared to 24 h *S. aureus* V. Moreover, *Cd14* and *Il1r2* were down-regulated in 24 h *S. aureus* L compared to untreated controls (Table 1 and Supplemental Table S1).The chemokines CXCL9 and CXCL10 are known to promote protective immune response during infections [67]. Recently, CD14 inhibition is known to efficiently attenuate early inflammatory response in bacterial sepsis [68]. IL1R2 is the well-known receptor of pro-inflammatory cytokines IL-1 alpha and IL-1 beta [69]. Taken together, as additional genes involved in inflammatory cascade (e.g. *Cxcl9*, *Cxcl10, Il1r2* and *Cd14* as discussed above) are differentially expressed with 24 h *S. aureus* L treatment, this could have a significant effect in reducing inflammation by L treatment in comparison to V treatment and untreated controls.

The glycerolipid metabolism pathway was strikingly reported only with L treatment comparison. Recent studies have shown that the disturbances in lipid metabolism, particularly those involving the components of GL/FFA cycling, are strongly associated with diseases related to the metabolic syndrome, inflammation and the pathogenesis of some cancers [70]–[72]. Also, many agents interfering with glycerolipid metabolism have been successfully used as additives or to treat several infections including toxic shock syndrome caused by *S. aureus* [73]–[76]. Thus, the involvement of glycerolipid pathway only with L treatment could have significance with its unique mode of action in treating staphylococcal infections. However, the exact significance of L treatment associated with glycerolipid pathway requires further elucidation.

The current study increases our understanding of the interplay between bacterial virulence and compensatory host response, and the impact of antibiotic treatment on this interplay. The *in vivo* evaluation of bacterial pathogenesis and host response is novel. The findings of this study help to unravel the complex relationship between host and pathogen in MRSA infection. Limitations include the fact that this study looked only at alpha hemolysin and PVL. We also studied only few cytokines based on the reported involvement of L and V on these cytokines [44], [45]. However, several other cytokines, both pro- and anti-inflammatory cytokines, might be associated with blood stream infection and we were not able to include all these cytokines in this study. For example, the serum level of an anti-inflammatory cytokine IL-10 is shown to be associated with mortality in severe sepsis [77] and recently in *S. aureus* bacteremia patients [78]. This warrants for the expansion of the cytokine panel in similar future studies. Based on the fact that the bacteria are demonstrable in murine blood stream within 2 hour post i.p. infection [35], we administered antibiotics 2 hour post infection. However, we recognize the fact that changing the time of introducing the antibiotic therapy could have a significant effect on cytokine and gene expression profile as is shown for its effect in mortality in patients with sepsis by Kumar A et al [79].

In summary, this study has provided the evidence that protein synthesis inhibitors like L could be superior in treating MRSA blood stream infections than the cell wall acting antibiotics. The superiority of protein synthesis inhibitors could be partly attributed to its better effect on lowering down the *in vivo* level of important staphylococcal toxins like alpha hemolysin and PVL as well as to its better immunomodulatory effects.

## Supporting Information

Table S1 **Genes differentially expressed in each comparison.**
(XLSX)
